# In Situ Synchrotron Diffraction Analysis of Zn Additions on the Compression Properties of NK30

**DOI:** 10.3390/ma12233935

**Published:** 2019-11-28

**Authors:** Domonkos Tolnai, Marie-Anne Dupont, Serge Gavras, Klaudia Fekete-Horváth, Andreas Stark, Norbert Schell, Kristián Máthis

**Affiliations:** 1 Institute of Materials Research, Helmholtz-Zentrum Geesthacht, Max-Planck-Straße 1, D 21502 Geesthacht, Germanysarkis.gavras@hzg.de (S.G.); andreas.stark@hzg.de (A.S.); norbert.schell@hzg.de (N.S.); 2 Nuclear Physics Institute of the AVCR, Řež 130, 250 68 Řež-Husinec, Czech Republic; fekete@karlov.mff.cuni.cz; 3 Department of Physics of Materials, Charles University, Ke Karlovu 5, Prague 2. 121 16 Prague, Czech Republic; mathis@met.mff.cuni.cz

**Keywords:** magnesium alloys, zinc addition, neodymium, Mg–Nd–Zn alloys, deformation behavior, in situ synchrotron radiation diffraction, acoustic emission

## Abstract

In situ synchrotron radiation diffraction was performed during the compression of as-cast Mg–3Nd–Zn alloys with different amounts (0, 0.5, 1, and 2 wt %) of Zn addition at room temperature. During the tests, the acoustic emission signals of the samples were recorded. The results show that the addition of Zn decreased the strength of the alloys but, at the same time, increased their ductility. In the earlier stages of deformation, twin formation and basal slip were the dominant deformation mechanisms. The twins tended to grow during the entire compression stage; however, the formation of new twins dominated only at the beginning of the plastic deformation. In order to accommodate the strain levels, the alloys containing Zn underwent nonbasal slip in the later stages of deformation. This can be attributed to the presence of precipitates containing Zn in the microstructure, inhibiting twin growth.

## 1. Introduction

Owing to its low weight and high specific mechanical properties, Mg is an ideal candidate to substitute heavier materials in applications where corrosion is not relevant [1]. The sounding specific properties can be paired with sufficient absolute ones by conscious alloy design [2]. Furthermore, controlled corrosion and the biocompatibility of Mg alloys can be exploited in the field of degradable implant applications [3]. In the development of Mg alloys for structural applications, one of the aims is to increase the strength of the materials. This can be accomplished by adding alloying elements to introduce secondary-phase particles or by thermomechanical processing to obtain recrystallization and the refinement of the microstructure. The addition of Zn and Rare Earth (RE) metals to Mg is an effective way of improving the mechanical property profile of Mg. This combination increases the peak strength and improves the castability and ductility of the material [4,5,6,7]. Adding Nd as a low solid solubility (3.6 wt % [8]) RE is a cost-effective solution, because a low amount of alloying addition is sufficient to induce secondary-phase formation in order to improve elevated temperature strength [9,10]. The presence of Zn further decreases the solubility of Nd [11]. The stable intermetallic phase in the Mg–Nd system is the Mg_41_Nd_5_. However, under realistic casting conditions, the Mg_12_Nd metastable phase is formed [12]. Given that the presence of Zn stabilizes the metastable Mg_3_(Nd, Zn) phase [13], extensive research has been recently devoted to the Mg–Nd–Zn system, aiming to characterize the mechanical behavior of the alloys at ambient and elevated temperatures under different load conditions and fatigue [9]. Because Nd is also biocompatible, these alloys are considered to be prospective implant materials for future medical applications [14].

The fast acquisition times of synchrotron radiation sources enable the observation of microstructural changes, by diffraction or tomography, with a high temporal resolution. Observing the changes in the grain structure allows for the determination of the dominant mechanisms during the deformation of the sample [15,16]. The evolution of the diffraction patterns acquired during the experiment sheds light on the development of the grain structure, on the changes in texture, and on strain and strain anisotropy, which can be correlated with twinning, recovery, and recrystallization [17,18]. Coupling the in situ diffraction with acoustic emission enables the extension of the nicety of the research on deformation mechanisms [19]. The aim of this work is to investigate the effect of Zn addition on the room temperature compression properties of an NK30 alloy in as-cast condition.

## 2. Materials and Methods

The alloys were cast in a permanent mold using the indirect chill casting method, described in [20]. High-purity Mg ingots were melted in a furnace with electric resistance heating. The chamber of the furnace was filled with a protective atmosphere, a mixture of Ar and 2 vol % SF_6_. The alloying elements of Zn and Nd were added in pure form; subsequently, 0.5 wt % of Zn grain refiner was added to the melt. Under continuous stirring, the melt was kept at 720 °C for 10 min and then filled into a steel mold preheated to 660 °C. The mold contacting the melt was held isothermally for 5 min and then quenched in water at a rate of 10 mm s^−1^ until the melt was submerged. A total of four alloys were prepared with the nominal compositions of Mg3Nd, Mg3Nd0.5Zn, Mg3Nd1Zn, and Mg3Nd2Zn. These alloys will be referred to in the paper as NK30, NZK300, NZK310, and NZK320, respectively. The compositional analysis of the cast ingots was performed using X-ray fluorescence spectroscopy (XRF) and spark optical emission spectroscopy.

The sample preparation for the metallographic investigations consisted of grinding the samples on SiC paper and polishing them with an OPS solution. They were then etched with an acetic-picral solution for optical microscopy (OM). The grain size was measured by the line intercept method. The scanning electron microscopy investigations were performed using a Zeiss FEG-SEM Ultra 55 (Carl Zeiss AG, Oberkochen, Germany) equipped with a Hikari detector (EDAX, AMETEK, Inc., Berwyn, PA, USA). The TSL-OIM software package (EDAX, AMETEK, Inc., Berwyn, PA, USA) was used to analyze the electron backscatter diffraction (EBSD). The EBSD measurements of the compressed samples focused on an area of interest of 400 × 500 μm with a step size of 0.5 μm around the center of the deformed specimen to capture an area similar to the one measured during the diffraction analysis. The microstructures from the deformed samples are presented here with the compression direction parallel to the horizontal direction.

For the in situ compression experiments, cylindrical specimens with a diameter of 5 mm and a length of 10 mm were fabricated using the cast ingots. The in situ experiments to assess the synchrotron radiation diffraction were conducted at the P07 beamline of PETRA III, DESY (Deutsches Elektronen-Synchrotron, Hamburg, Germany). The monochromatic beam energy was set to 100 keV (λ = 0.0124 nm) with a cross section of 1 × 1 mm^2^. The diffraction patterns were recorded with a PerkinElmer 1621 flat panel detector with a pixel size of 200 μm^2^. The sample-to-detector distance was set to 1621 mm and was calibrated by measuring a LaB_6_ standard powder sample. The acquisition time for each pattern was 1 s. The specimens were compressed with a DIL 805A/D (TA Instruments, New Castle, DE, USA) dilatometer. The deformation rig was modified in order for the beam to pass only through the sample [21]. The specimens were compressed at room temperature with an initial strain rate of 1.0 × 10^−3^ s^−1^. The morphology of the Debye–Scherrer patterns was analyzed using the Fit2D software (ESRF, Grenoble, France) and converted into azimuthal angle time (AT) plots with the ImageJ software package (NIH, Bethesda, MA, USA).

The acoustic emission (AE) response of the samples was recorded using the Mistras PCI-2 acquisition board. A S9215 high-temperature (up to 650 °C) broadband AE sensor from Mistras Group, Inc. (Princeton Junction, NJ, USA). was mounted on the compression heads using vacuum grease and a metallic collar. The AE signal was amplified by 60 dB in the frequency range of 100–1200 kHz using a Mistras 2/4/6 preamplifier. The AE response was monitored with a so-called data-streaming regime, while a raw signal of 1 MHz sampling rate was recorded in a threshold-less mode during the deformation tests.

## 3. Results

### 3.1. Metallography

The actual alloy compositions of the investigated alloys measured with spark optical emission spectroscopy and X-ray fluorescence spectroscopy (XRF) are listed in Table 1.

The alloy compositions are reasonably close to the nominal values, taking into account the small size of the casting batch. Owing to the indirect chill casting, the microstructure is homogeneous throughout the solidified parts. The grain sizes measured on the optical microscopy images with the line intercept method are listed in Table 2.

**Figure 1:**
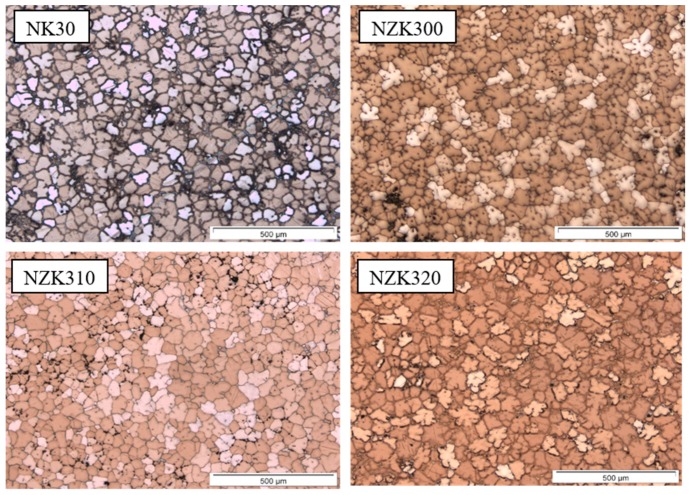
Optical micrographs of the investigated alloys.

As expected, the addition of alloying elements caused a decrease in the grain size. The effect of the addition of Zn is marginal and only becomes relevant above 0.5 wt %. The as-cast microstructures of the investigated alloys are shown in Figure 1.

**Figure 2:**
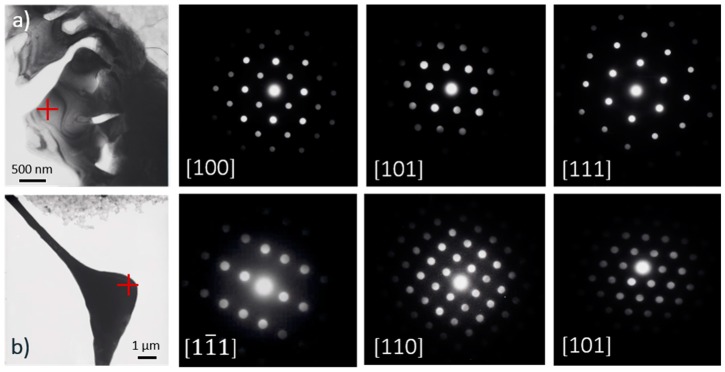
TEM results on the intermetallic particles in NZK 320 (**a**) Mg_3_(Nd,Zn) (FCC), (**b**) Mg_12_Nd (C-centered orthorhombic).

The binary NK30 alloy consists of equiaxed dendrites and intermetallic particles located at the grain boundaries. The addition of Zn does not change the microstructure of the NZK alloys. The initial intermetallic phase in the NK30 alloy is Mg_12_Nd. TEM investigations were conducted to determine the effect of Zn on the type of intermetallic particles (Figure 2).

The TEM results show that in the lower Zn-containing alloys, NZK300 and NZK310, the continuous intermetallic phase, the C-centered orthorhombic Mg_12_Nd, is present. In the case of NZK320, the additional amount of Zn gives rise to the formation of the FCC Mg_3_(Nd,Zn), which generates a lamella-like structure at these cooling rates.

### 3.2. In Situ Compression Experiments

**Figure 3:**
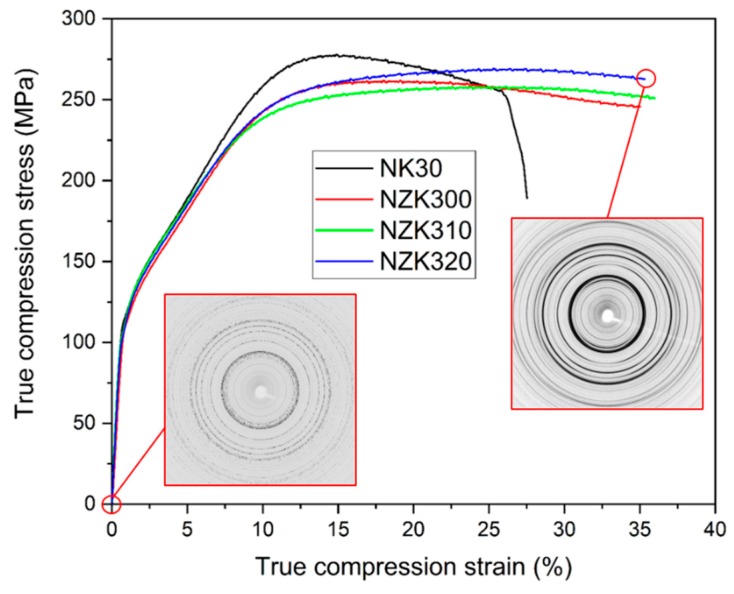
True stress–true strain curves obtained during the in situ compression experiments, with the diffraction patterns of NZK320 in the as-received state and before fracture.

The true stress–true strain curves obtained during the in situ deformation experiments are shown in Figure 3.

**Figure 4:**
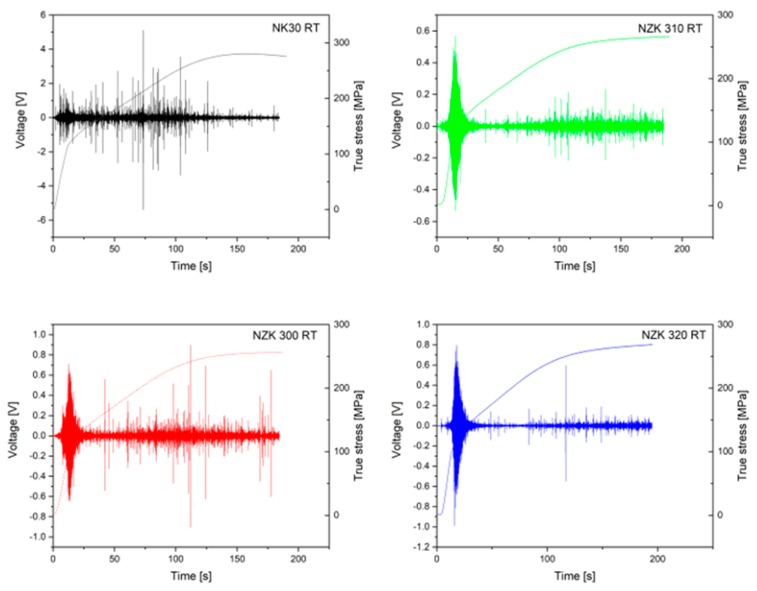
Acoustic emission signal, recorded during the compression of the samples.

The diffraction pattern at the beginning of the experiment consists of small individual diffraction peaks along the reflection ring, due to the small grain size. At the end of the experiment, a typical compression texture can be seen with continuous Debye–Scherrer ring sections. This can be attributed to the decrease in grain size during the deformation. The binary NK30 alloy exhibited the highest ultimate compression strength. The modification of the binary alloy with Zn caused a decrease in the overall strength of the alloy but increased the ductility. An inflection point can be observed on the curves approximately at 3% of strain in all the alloys tested. This indicates that twinning was the main deformation mechanism at the beginning of plastic deformation. From the diffraction patterns at the beginning and the end of compression, it can be concluded that the grain size decreased significantly. The acoustic emission signals of the alloys acquired during compression are shown in Figure 4.

The AE response for the binary and the Zn-containing alloys is different. The overall AE intensity level is higher for NK30 (note the difference in the *Y*-axis scale of the bottom graphs), and a strong AE burst is present during the entire straining period. Such bursts are connected with extension twinning [22]. In contrast, the NZK alloys have a characteristic “tear” AE response in the vicinity of the yield point, connected with the concurrent activity of twinning and basal slip, but only a small burst can be observed in the later stage of the deformation.

**Figure 5:**
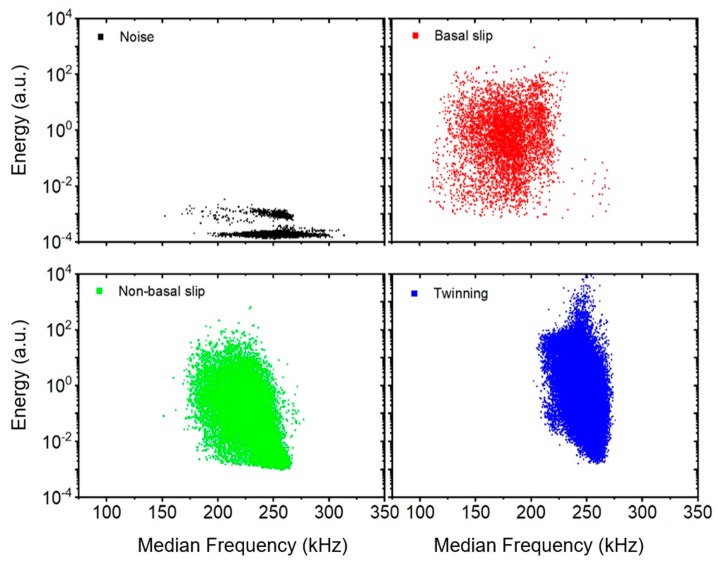
Energy–median frequency cross-plots for the acoustic emission (AE) events in the particular clusters–noise, basal slip, nonbasal slip, and twinning (the plots for NK30 room temperature testing are presented here; the distribution for other alloys and testing temperatures is similar).

**Figure 6:**
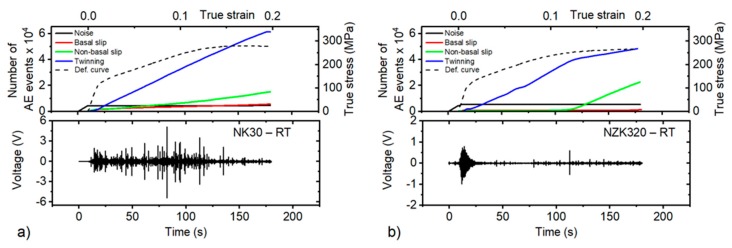
Time evolution of the number of AE events in the particular clusters and the corresponding stress–strain curve (dash line) and AE stream (bottom graph) for (**a**) NK30 deformed at room temperature; (**b**) NZK320 deformed at room temperature.

In magnesium alloys, several deformation mechanisms (such as the dislocation slip and twinning) are concurrently active, and the resultant AE is a convolution of the signal from many emitting sources. Therefore, the conventional hit-based recording mode, where the AE signal is parametrized using a preset threshold level and a hit definition time, does not enable the separation of the “twinning” and “dislocation” signals [23]. In order to identify the dominant mechanism in different stages of the straining period, the adaptive sequential k-means (ASK) cluster analysis was applied to the AE data streams [23]. The details of the ASK clustering algorithm applied to the deformation behavior of Mg alloys were discussed in our previous papers [24,25]. The continuously recorded data were sectioned into consecutive individual realizations (“frames”) containing 2048 samples. The frames were then clustered according to the similarity of their power spectral density (PSD) functions G(f). The Kullback–Leibler distance was used to measure the similarity of the shapes of normalized PSDs. Based on the time of appearance and mutual distributions of physical parameters (amplitude, energy, frequency range) of the cluster elements (Figure 5), a dominant AE source mechanism can be associated with each cluster. Finally, a time (strain) evolution of a cumulative number of elements in particular clusters can be plotted (Figure 6), indicating which mechanism is dominant at a given stage of the straining period. However, the concurrent activity of other mechanisms, with a higher nonbasal slip, is not excluded. Because the results for NZK300, 310, and 320 are almost identical, only the ASK analyses for NK30 and NZK320 are presented below.

The ASK analysis identified four clusters. The first one, with a low energy level and a broad frequency range (Figure 5), appeared before the deformation test was launched (Figure 6) and is naturally associated with the background noise. Two clusters related to dislocation slip exhibiting low to medium energy levels were identified. The first one emerged when stress levels were low, immediately after the beginning of the loading period, which is in good agreement with the low critical resolved shear stress of basal slip. The second one appeared when stress levels were higher, which is characteristic of nonbasal slip (this method does not allow for a differentiation between the different nonbasal slip systems). The shifted frequency range with respect to the basal slip cluster is determined by the fact that, at a later stage of deformation, the mean free path for dislocation slip is shorter, and the stress field of previously generated dislocations delays the dislocation movement. Therefore, the dislocation clusters are typically shaped as a “tear”. The high amplitude signals from clusters originating in twinning events fell into a narrower frequency range. It should be emphasized that only twin nucleation and propagation (growth in length) emit detectable AE, whereas twin thickening does not [22].

**Figure 7:**
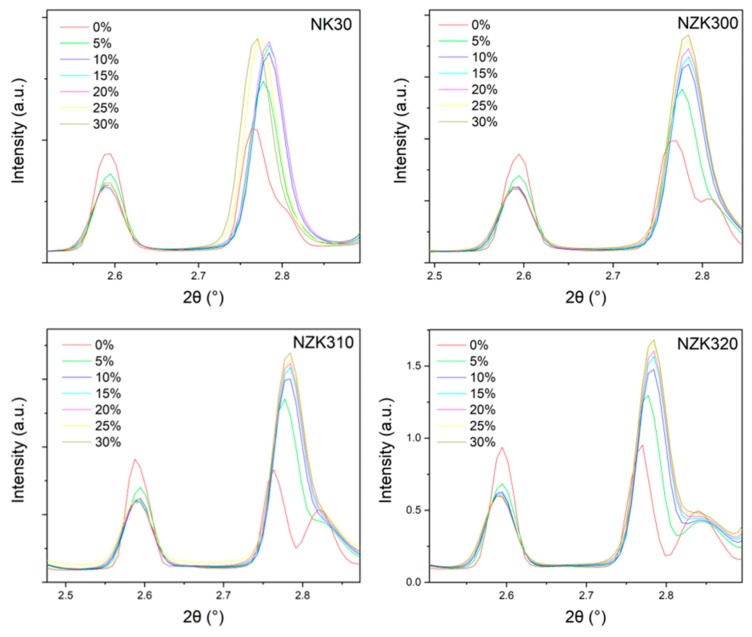
Changes in the intensity of the 10.0 and the 00.2 peaks during compression.

Twinning dominates the deformation of both NK30 and NZK320. However, some differences can be observed in the particular cluster evolution (Figure 6a,b). To begin with, more AE events originated in twinning for NK30 than in twinning for NZK320. On the other hand, the activity of nonbasal slip is much higher for the NZK320 alloy. This can be associated with the need to activate the nonbasal slip (particularly the second-order pyramidal slip system) for the accommodation of the strain, because the twinning is hindered by the presence of solutes in the Zn-containing alloys. The extension twinning further results in a change of texture, which can lead to the rotation of several grains to orientations with high Schmid factor for basal slip. In line with this, a number of elements in basal slip clusters increased above approximately 10% of strain, and the effect was once again larger for NK30, owing to the larger twinned volume. The changes of the 10.0 and the 00.2 peaks of Mg during compression are shown in Figure 7.

**Figure 8:**
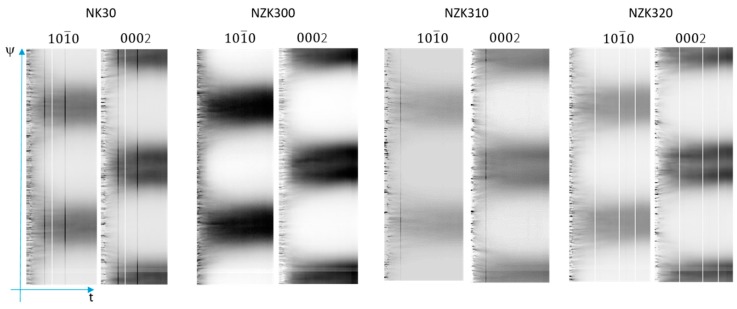
Azimuthal angle time plots of the 10.0 and 00.2 lines of Mg during the compression tests.

The coincident changes in the intensity of the 10.0 and 00.2 peaks indicate twinning. As the grains twin, there is a reorientation of 86° that is close to the 90° angle between these two planes. When twinning occurs, the 10.0 peak loses intensity, while the 00.2 peak becomes more intense. The behavior of all four alloys is similar. There is a continuous change, however, with the largest increment occurring at the beginning of the plastic regime and continuing with smaller changes throughout the compression period. This indicator is nonetheless sensitive to the twinned volume, which means that the initiation of new twins, or the growth of the existing ones, cannot be distinguished. The azimuthal angle time plots of the 00.2 and the 10.0 diffraction lines of Mg are shown in Figure 8.

At the beginning of the compression, straight timelines originating from the individual crystals can be observed in the elastic regime. Once the deformation passes the yield point, these lines start to deviate. In the NK30 binary alloy, at the beginning of the deformation, the appearance and disappearance of the timelines can be caused by twinning at approximately 3% of strain. This was also visible on the compression curves. As the deformation continues, the timelines bend (grain rotation) and broaden (dislocation formation). In the end, a typical compression texture takes shape. A similar trend can be observed in the NZK alloys. Twinning is noticeable at the beginning of the plastic regime, but the number of disappearing and appearing timelines is lower than in the binary alloy. Afterwards, mostly grain rotation and dislocation formation can be observed.

### 3.3. EBSD Analysis

**Figure 9:**
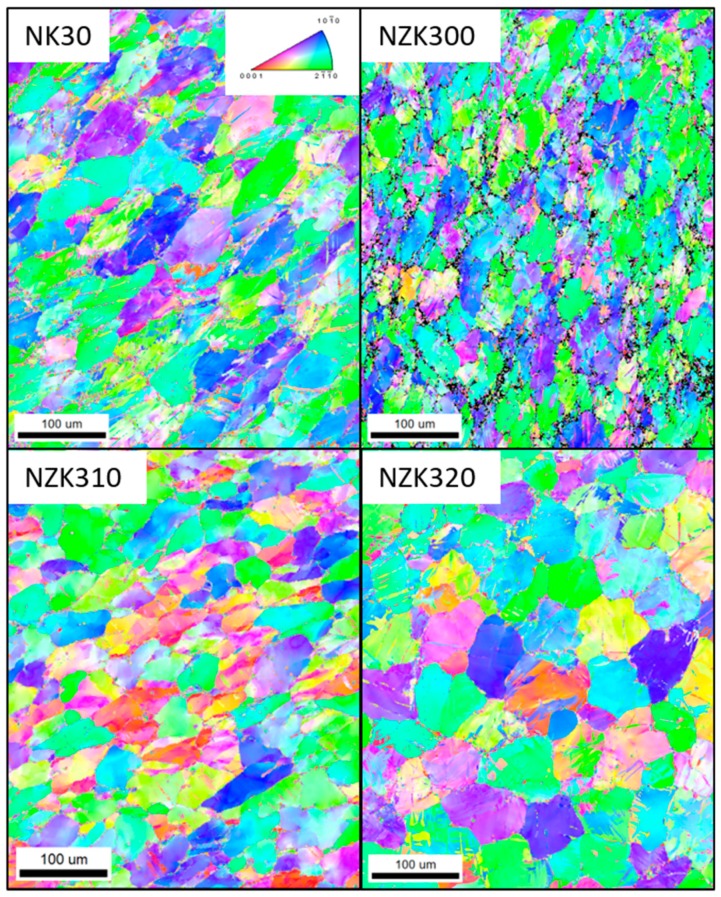
Postmortem electron backscatter diffraction (EBSD) maps of the investigated samples.

The results of the postmortem EBSD analysis at 30% of deformation are shown in Figure 9.

**Figure 10:**
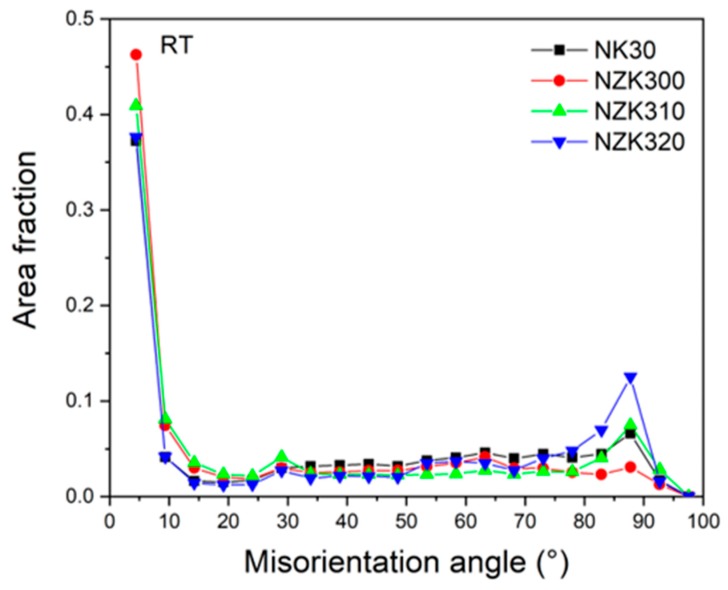
Misorientation angles obtained from the postmortem EBSD results.

On the Inverse Pole Figure (IPF) maps, twins can be observed in all the alloys, but their amount is lower than the one suggested by the diffraction results. It is likely that the twinning process resulted in the transformation of larger regions of the grain structure. Besides twinning, the results also show the formation of low-angle grain boundaries, which can be attributed to the dislocation buildup in the grains. In the ternary alloys, the process is similar to the one found in the binary alloys, although the former’s microstructure contains more twins than that of NK30. This can be attributed to the presence of precipitates, as they can hinder the growth of the existing twins. The misorientation angle distribution is shown in Figure 10.

The misorientation angle distribution shows a peak at 86° in all the alloys, which also indicates that the grains underwent twinning during the deformation period.

## 4. Discussion

The metallographic characterization of the alloys shows that the addition of Zn did not have a significant influence on the grain size. This suggests that the addition of Zr has a greater grain-refining effect, which cannot be further improved significantly by Zn. The intermetallic phase on the grain boundaries is the Mg_12_Nd phase [26], a metastable phase formed under moderate cooling rates. The Mg_41_Nd_5_ stable phase only emerges with long periods of heat treatment [12]. Previous studies show that the presence of Zn stabilizes the Mg_3_(Nd, Zn) phase [27]. This phase was found in the 2 wt % Zn containing the NZK320 alloy, which places the phase field boundary between 1 and 2 wt % of Zn addition.

The addition of Zn increased the ductility but, at the same time, decreased the ultimate compressive strength. Due to the specificity of the tests, the number of tested samples is insufficient to draw any conclusion regarding their mechanical properties. At approximately 3% of strain, an inflection point appears on the deformation curves. In the case of materials with an HCP crystal structure, this can usually be attributed to twinning, because at room temperature, the activation energy of nonbasal slip is significantly higher than that of twin formation. The latter can be seen on the postmortem EBSD results, and the remaining twins can also be observed. Moreover, the misorientation plot indicates that there was twin formation and growth during the deformation process. The incorporation of in situ synchrotron radiation diffraction and acoustic emission in the compression setup allows for a more detailed analysis of the deformation mechanisms. The diffraction results show a sudden increase in the twinned volume at the beginning of deformation that is usual in the case of Mg at ambient temperatures. As the compression continues, the twinned volume increases at a slow pace. The AE technique is sensitive to the twin formation but not to the growth. There is a signal of twin initiation at the beginning of the experiment but not at later stages. This signal, together with the diffraction results, means that twin formation is initially the main deformation mechanism and that, later, the existing twins tend to grow. The diffraction results also show that twin growth is accompanied by grain rotation and the broadening of the ψ angle distribution on the diffraction patterns at these later stages, owing to the dislocation buildup within the grains. At higher strain levels, the AE analysis clearly shows that in the case of a hindered twin boundary movement, the activation of the nonbasal slip is necessary for strain accommodation. The signal is higher for the alloy containing Zn, suggesting that the precipitates in the modified alloys hinder twinning.

## 5. Conclusions

The NK30 alloy exhibited higher UCS, but the addition of Zn increased the ductility of the alloys. Alloying with 2 wt % gave rise to the formation of the Mg_3_(Nd,Zn) phase, in addition to the Mg_12_Nd phase. This suggests a phase field boundary located between 1 and 2 wt % of Zn addition. The deformation mechanisms of the four alloys are very similar. At the early stages of plastic deformation, a large number of twins were formed. The twinned volume grew continuously during compression; however, this was likely due to the growth of the existing twins. Twinning was accompanied by basal slip in all the alloys, but, in the later stages, nonbasal slip became active in the alloys containing Zn. This can be due to the Zn-containing precipitates within the grains, which might have hindered twin growth.

## Figures and Tables

**Table 1:** Chemical compositions of the alloys.

Alloy (wt %)	Nd wt % (XRF)	Zn wt % (Spark Analyzer)
NK30	3.7	-
NZK300	3.7	0.48
NZK310	3.7	11
NZK320	3.7	1.9

**Table 2:** Average grain size of the alloys prior to compression.

Alloy (wt %)	Grain Size (μm) ± SD
NK30	57.7 ± 1.7
NZK300	55.4 ± 3.4
NZK310	49.7 ± 3.8
NZK320	49.5 ± 6.7
